# SPIONs/3D SiSBA-16 based Multifunctional Nanoformulation for target specific cisplatin release in colon and cervical cancer cell lines

**DOI:** 10.1038/s41598-019-51051-w

**Published:** 2019-10-10

**Authors:** B. Rabindran Jermy, Munther Alomari, Vijaya Ravinayagam, Sarah Ameen Almofty, Sultan Akhtar, J. Francis Borgio, Sayed AbdulAzeez

**Affiliations:** 1 Department of Nano Medicine Research, Institute for Research and Medical Consultations, Imam Abdulrahman Bin Faisal University, Dammam, 31441 Saudi Arabia; 2 Department of Stem Cell Biology, Institute for Research and Medical Consultations, Imam Abdulrahman Bin Faisal University, Dammam, 31441 Saudi Arabia; 3 Department of Biophysics Research, Institute for Research and Medical Consultations, Imam Abdulrahman Bin Faisal University, Dammam, 31441 Saudi Arabia; 4 Department of Genetic Research, Institute for Research and Medical Consultations, Imam Abdulrahman Bin Faisal University, Dammam, 31441 Saudi Arabia

**Keywords:** Diagnostics, Cancer therapy

## Abstract

Multifunctional nanomaterials can be used for dual applications: drug delivery as well as in bioimaging. In current study, we investigated potential use of silica based supports; 3D cage type SiSBA-16 (S-16), monodispersed hydrophilic spherical silica (HYPS) and mesocellular foam (MSU-F) for cisplatin (Cp) delivery. To obtain magnetic resonance characteristics, 10 wt% iron oxide was loaded through enforced adsorption technique. For pH stimuli responsive release of Cp, 10 wt%SPIONs/S-16 was functionalized with 3-(Aminopropyl)triethoxysilane (A) and poly acrylic acid (PAA) termed as 10 wt%SPIONs/S-16-A-Cp and 10 wt%SPIONs/S-16-APAA-Cp. By TEM analysis, the average diameter of the SPIONs was found to range between 10–60 nm. VSM analysis showed saturation magnetization over S-16, HYPS and MSU-F were in the following order: 10 wt%SPIONs/HYPS (4.08 emug^−1^) > 10 wt%SPIONs /S-16 (2.39 emug^−1^) > 10 wt%SPIONs/MSU-F (0.23 emug^−1^). Cp release study using dialysis membrane in PBS solution over 10 wt%SPIONs/S-16 nanoformulations showed highest cumulative release (65%) than 10 wt%SPIONs/MSU-F-A-Cp (63%), 10 wt%SPIONs/HYPS-A-Cp (58%), and Cp-F127/S-16 (53%), respectively. 10 wt%SPIONs/S-16-A-Cp and 10 wt%SPIONs/S-16-APAA-Cp were evaluated for *in vitro* target anticancer efficiency in human cancer cell lines (colon cancer (HCT 116), cervical cancer (HeLa)) and normal cells (Human embryonic kidney cells (HEK293) using MTT and DAPI staining. 10 wt%SPIONs/S-16-A-Cp treated Hela and HCT116 cancerous cell lines showed significant control of cell growth, apoptotic activity and less cytotoxic effect as compared to Cp and 10 wt%SPIONs/S-16. Target specific Cp release in the cells shows that 10 wt%SPIONs/S-16-A-Cp can be easily upgraded for magnetic resonance imaging capability.

## Introduction

Cisplatin (Cp) is a therapeutic drug used for the cancer therapy. The cytotoxic action of drug is limited by adverse side effects such as developing resistance, nephrotoxicity, neuropathy, dose toxicity, hearing loss etc. Currently, several nanocarriers such as polymers, structured silicas, liposomes are reported to facilitate controlled and sustained drug release of Cp for longer time period. For instance, Cp/nanofiber based on poly caprolactone and chitosan prepared through electrospinning technique was reported to treat cervical cancers^1^. Biocompatible copolymer poly (lactic-co-glycolic acid) has been reported to encapsulate high content of Cp (about 70%) through electrohydrodynamic atomization technique. Varying the Cp to polymeric ratio reported to generate small sized nanoparticles critical for less burst (14%) Cp release^2^. Similarly, a reverse multi-step microemulsion technique reported to increase the solubility of Cp source in the presence of potassium chloride^3^. Recently, we have shown that the hierarchical structured mesosilicalite capability to carry cisplatin^4^.

A combination of therapeutic compounds with tumor imaging agents has been reported to improve the treatment efficacy and limit the side effects due to onsite drug delivery^5^. The biomedical usage of SPIONs are more advantages due to FDA approved particles for clinical use, shows intrinsic magnetic characteristics and are flexible for modification involving drug molecules. Such magnetic nanosilicas has already been shown to have the potential in clinical trials, phase I/II^6^. Smart polymers were also reported to responds to the external magnetic field as well to pH and temperature changes^7^. Magnetic Fe_3_O_4_ based mesoporous silica such as SBA-15 (p6mm) and thermal stimuli copolymer coated magnetic mesosilica (MMSN@P(NIPAM-co-MAA) were also reported to be effective for multifunctional drug delivery applications^8,9^. Dual imaging (fluorescence-magnetic) diagnostic tool with amine functionalized iron oxide/SBA-16 nanocomposite was found to be a suitable carrier for large protein molecules^10^. Curcumin loaded in the SPIONs coated with hyaluronic acid (fluorescent dye) was found to be effective for MRI and fluorescent imaging studies^11^. A biodegradable silica/iron oxide nanocomposite with saturation magnetization of 1.65 emu/g was reported with mesopores ranging 20–60 nm for protein Ferritin delivery (cargo size >15 nm)^12^. The nanocomposite exhibited both pH stimuli (pH 5.0 > pH 7.4) and magnetically driven (magnetic to thermal energy) protein release. Zhu *et al*.^13^ has reported the synthesis of Fe_3_O_4_/mesoporous silica composite through core-shell technique to deliver Cp. The composite has shown a well dispersed iron oxide nanoparticles (~85 nm) with magnetization property of 37 emu/g. pH responsive cisplatin release was observed in A549 and MCF-7 cell line^14^. Magnetically separable Fe_3_O_4_@SBA-15 nanocomposite was reported using salt of FeCl_2_ and FeCl_3_ under nitrogen degassing atmosphere followed by ammonia addition^15^.

However, cost effective multimodal bioimaging carrier for Cp is still limited. Developing magnetically active nanocarrier and functionalizing drugs in a single entity is difficult and requires multi-step protocols^8,9^. Recent study showed only 5–10% of drug reaches the tumors (bioavailability), which is equal to the conventional drug (without nanocarrier) efficiency^16^. Considering the cost of nanodrug carriers development and mode of transport technology, it is too far from commercial application both from the point of target efficiency and cost of technology development. The objective of the present study is to optimize a new nanoformulation involving 3-D cage type S-16, spherical HYPS and mesocellular foam nanocarriers. SPIONs were impregnated by simple enforced impregnation technique. For pH stimuli Cp release, nanocarrier was functionalized with 3-(Aminopropyl) triethoxysilane (A), and polyacrylic acid (PAA). The presence of high surface area and magnetically active SPIONs over S-16 exhibited high Cp release and targeted anticancer activities in *in-vitro* against cancer cell lines such as colon cancer (HCT 116) and cervical cancer (HeLa) cells, prove harmless nature of nanoformulation on normal human embryonic kidney cells.

## Material and Methods

Mesocellular foam (MSU-F) was purchased from Sigma Aldrich, while HYPS was purchased from Superior silica, USA. S-16 was synthesized based on our earlier reported literatures^4,17^. In brief, 6 g of F127 (Sigma) was dissolved in 290 g of H_2_O through stirring for 2 h. Then 12.6 g of 35 wt% HCl and 19.2 g of nBuOH was added at once and stirred for 1 h. Later, 29 g of TEOS (Aldrich, 98%) was added and again stirred at 40 °C for 24 h. The solution was hydrothermally heated at 100 °C for 24 h. Then the mixture cooled, filtered, washed and dried in air for 5 h, then dried at 100 °C overnight. Then template was removed by calcination at 550 °C for 6 h.

### Preparation of 10 wt%SPIONs/nanosupport (S-16, HYPS and MSU-F)

The support S-16, HYPS and MSU-F were predried at 120 °C for 24 h. 10 wt% of iron oxide was loaded over each support by enforced adsorption technique. In case of 10 wt%SPIONs/S-16, 0.72 g of iron (III) nitrate nonahydrate was dissolved in 80 ml water for 10 min. S-16 (1 g) was added and stirred overnight. Then aqueous mixture was dried at 120 °C for 12 h and finally calcined at 550 °C for 2 h.

### Preparation of 10 wt%SPIONs/S-16-APAA-Cp

The 10 wt%SPIONs/S-16 composite (1 g) was then functionalized with 1.5 ml of 3-Aminopropyl)triethoxysilane silane (A) using anhydrous toluene (100 ml) solution mixture under refluxing condition in argon atmosphere. The solution mixture was then filtered using what paper in glass funnel and then vacum dried for 3 h to produce 10 wt%SPIONs/S-16-A. The sample was then wrapped with 40 mg of polyacryic acid (PAA) by dispersing in 40 ml of DMF under refluxing condition at 140 °C for 2 h. The content was then cooled down, filtered, washed with methanol (5 ml) thrice and dried at room temperature to form 10 wt%SPIONs/S-16-APAA. The Cp loading was carried out by dissolving 30 mg of Cp with 600 mg of 10 wt%SPIONs/S-16 or 10 wt%SPIONs/S-16-APAA in normal saline solution (NSS). The mixture was stirred overnight in an ice cooled dark environment and then filtered, washed, and dried at room temperature to obtain the final product. The Cp content determined to be 0.12 mmol/g of nanocarrier^4^.

### Characterization of the prepared products

X-ray diffraction pattern for SPIONs/silica nanoformulations were analyzed using bench top Rigaku Multiplex system (Rigaku, Japan). Textural characteristics involving surface area and pore size distributions of parent and formulations were measured using an ASAP-2020 plus (Micromeritics, USA). The samples were pretreated before measurements at 523 K for 2 h. The surface area and pore sizes were calculated using BET and BJH technique. Surface area evalulated by considering adsorption values of linear plots for BET. Pore volume obtained at p/p0 = 0.98, while micropore surface area was calculated based on t-plot technique. Cp functional groups were identified using FT-IR spectroscopy equipped with attenuated total reflectance (ATR) (Perkin Elmer, USA). The surface features of the synthesized materials were characterized by scanning electron microscopy and transmission electron microscopy. For SEM (FEI, Inspect S50, Czech Republic), the prepared powder was dispersed onto doubled sided tape holder and examined under 20 keV. Depending on the morphology, different magnifications were chosen to capture the representative features of the specimens. TEM samples were prepared by dispersing a small amount of sample in ethanol and deposited onto TEM grid. The grids were examined in a TEM instrument; FEI, Inspect S50, Czech Republic at working voltage of 80 kV. Several images were acquired to measure the particle size and calculate an average size. The particle size was measured using Gatan digital micrograph software. The results are displayed in the form of size histogram for each prepared sample.

### Drug adsorption study

In Cp adsorption study, the SPIONs loaded formulations: 10 wt%SPIONs/S-16, 10 wt%SPIONs/HYPS and 10 wt%SPIONs/MSU-F (600 mg) were mixed separately with 30 mg of Cp in 10 ml of saline solution under ice cooled dark environment. After overnight stirring, the solution mixture was filtered, washed with 15 ml of normal saline solution. Then the adsorbed Cp was calculated using UV-visible spectroscopy (JASCO, Japan) at 208 nm based on the equation.

$${\rm{Adsorption}}\,( \% )=({X}_{0}-{X}_{1})/{X}_{0}\times 100$$

where X_0_ and X_1_ corresponds to initial Cp (mg) -free Cp (mg).

### Drug release study

The cumulative Cp release was studied using the prepared nanoformulations: 10 wt%SPIONs/S-16, 10 wt%SPIONs/HYPS and 10 wt%SPIONs/MSU-F. Drug delivery was performed by immersing 30 mg of drug formulations in the cellulose membrane dialysis tubing bag in 50 ml of phosphate buffered saline (PBS) at pH 5 and pH 7 (for selected sample). The release condition was performed under constant temperature at 37 °C. At regular time interval, specific volume of solution was removed (10 ml), replaced with fresh PBS solution and analyzed using UV-visible spectroscopy.

### *in-vitro study on* HeLa, HCT116 and HEK-293 cells

Based on the drug release study, we chose the best, 10 wt%SPIONs/S-16 nanoformulation and tested in *in-vitro* study on cancerous cells as following groups:

Group-I- Cancer cells treated S-16

Group-II = Cancer cells treated with SPIONs

Group-III = Cancer cells treated with 10 wt%SPIONs/S-16

Group-IV = Cancer cells treated with Cp

Group-V = Cancer cells treated with 10 wt%SPIONs/S-16-APAA-Cp

Group-VI = Cancer cells treated with 10 wt%SPIONs/S-16-A-Cp

Antitumor effect of 10 wt%SPIONs/S-16-A-Cp, 10 wt%SPIONs/S-16-APAA-Cp and Cp were compared on cancer cells lines, HeLa (Cervical cancer; ATCC^®^ CCL-2™) and HCT 116 (Colon cancer; ATCC^®^ CCL-247™) and on non*-*malignant cell line HEK-293 (Human embryonic kidney cells, ATCC^®^ CRL-1573™). Cells were grown in RPMI (HyClone, GE Healthcare, Chicago, USA) medium with 10% fetal calf serum (HyClone, GE Healthcare, Chicago, USA) and 1% penicillin/streptomycin solution (Thermo Fisher, Waltham, USA) at 37 °C in 5% CO_2_ incubator (Heracell 150i, Thermo Scientific, Waltham, MA). The cells were seeded at the rate of 1 × 10^4^ cells/each well of a 96 well plate (Thermo Fisher, Waltham, USA). At 24 hours of culture, the cells were treated with varied concentration of nanoformulated drugs, along with the vector in which the drugs were solubilized. Upon further culture for another 24 hours, the cells were subjected to end point cell survival assay using MTT (3-(4,5-Dimethylthiazol-2-yl)-2,5-Diphenyltetrazolium Bromide) dye reduction test.

### MTT assay

To each of the drug treated and control cell culture wells of 96 well plate, 20 µl of MTT (10 mg/ml) was added in drug treated cells and control cell in 96 well plate. Inncubated for for 4 h. The cells were washed with PBS to remove dye. Formazan dye formed was solubilized by adding 150 µl of MTT solvent using plate shaker (15 mins). Optical density(OD) measurement of solubilized dye was observed at 590 nm using a multiplate reader (Tecan Infinite^®^ 200 PRO, Switzerland). The OD was compared and calculated against control and expressed as percentage of cell survival. The resulted data was blotted in excel and calculated the LC50 using the equation of the logarithmic trendline. The p value and standard deviation were measured by one-way analysis of variance (ANOVA) in GraphPad prism software based on triplicates experiments (p values < 0.05 were considered significant).

### DAPI staining

Cells viability of treated and control cells were tested by DAPI staining. Pretreated HeLa and HCT116 cells with Cp, 10 wt%SPIONs/S-16-A-Cp, 10 wt%SPIONs/S-16-APAA-Cp or vehicle control were washed with 1x PBS then fixed with ice cold Methanol for 5 minutes then stained with DAPI (Sigma Aldrich) for 10 minutes in the dark. Cells were washed again with 1x PBS. The wells were dried up and mounted with ProLong ® Gold Antifade reagent (Thermo Fisher, Waltham, USA). The images were developed by ZEN software using confocal microscopy (Zeiss, Oberkochen, Germany).

### Statistical analysis

MTT cell cytotoxicity assay results were subjected to One-way ANOVA followed by Dunnett’s post hoc test of GraphPad Prism software (GraphPad, La Jolla, CA) on three independent set of experiments conducted in triplicates. *p* values < 0.05 were considered significant.

## Results and Discussion

**Figure 1:**
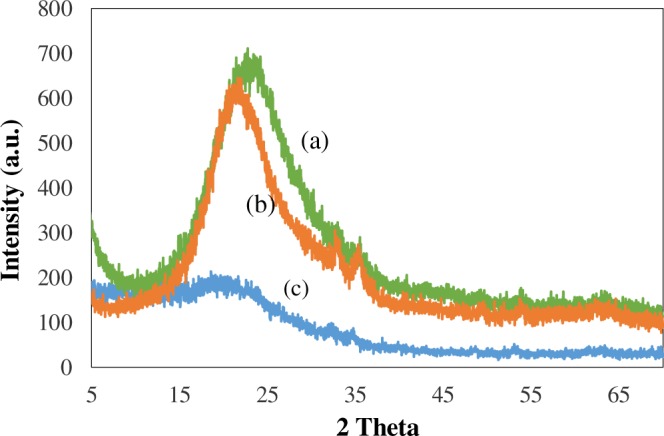
X-ray diffraction pattern of (**a**) 10 wt%SPIONs/S-16 (**b**) 10 wt%SPIONs/HYPS and (**c**) 10 wt%SPIONs/MSU-F.

Figure 1(a–c) shows the XRD diffraction patterns of SPIONs loaded on different structured silicas S-16, HYPS and MSU-Foam. 10 wt% of SPIONs loadings was carried out using enforced adsorption technique. The presence of characteristics broad peak due to amorphous siliceous framework was observed between 15–30°. The presence of Fe_3_O_4_ was expected to be observed at 2 theta value of 35.45°. In case of 10 wt%SPIONs/S-16 and 10 wt%SPIONs/HYPS, the presence of weak diffraction peaks was observed, which corresponding to cubic structure of Fe_3_O_4_ (magnetite, PDF # 88-0866) (Figure 1a,b). The presence of such weak peaks with increased broadness is due to small nanosized Fe_3_O_4_ indicating lack of crystallization inside cubic cage and spherical nanopores (nanocarriers). The presence of broad peak in the spectra is in good correlation with the high surface area of S-16 that reflects finer particles dispersed on the 3D cage type pores (Table 1). However, the peaks intensity at 35.4°, 43.1°, 53.4°, and 62.6° increased over 10 wt%SPIONs/MSU-F corresponding to (311), (400), (422), and (440) (Fig. 1c). This shows that surface modification of Fe_3_O_4_ particles tends to change depending on the nature of structured silica. In case of Cp, crystalline phase of Pt complex was clearly observed with characteristics diffraction patterns (Fig. S1a). In case of Fe/S-16-APAA-Cp, a broad diffraction peak of amorphous phase of silica was observed. The absence of Cp peaks in the diffraction patterns shows the transformation of crystalline to amorphous phase of Cp due to confinement in the cubic pores of SBA-16, beneficial to enhance the solubility of Cp (Fig. S1b).

**Table 1:** Textural characteristics of (a) S-16 (b) 10 wt%SPIONs/S-16, (c) HYPS, (d) 10 wt%SPIONs/HYPS, (e) MSU-F, and (f) 10 wt%SPIONs/MSU-F.

Sample	Fe_3_O_4_ Loading (wt%)	BET Surface area (m^2^/g)	BJH adsorption cumulative surface area (m^2^/g)	t-plot micropore surface area (m^2^/g)	Pore volume (cm^3^/g)	PD (nm)
S-16	—	988	590	340	0.69	3.3
SPIONs/S-16	10	471	297	127	0.37	3.8
HYPS	—	170	85	76	0.35	8.3
SPIONs/HYPS	10	56	54	2	0.21	15.5
MSU-F	—	540	554	21	2.28	40.9
SPIONs/MSU-F	10	140	134	—	1.41	40.2

The surface area and pore size distributions of parent and SPIONs loaded samples were analyzed using nitrogen adsorption technique. Fig. S2(a–f) shows the isotherms for (a) S-16 (b) 10 wt%SPIONs/S-16, (c) HYPS, (d) 10 wt%SPIONs/HYPS, (e) MSU-F, and (f) 10 wt%SPIONs/MSU-F. Textural results are presented in Table 1. The isotherm pattern of S-16 shows a typical spinodal hysteresis pattern for ink bottle type of pores. In case of SPIONs loading over S-16, a significant decrease observed in the textural characteristics. Specifically, a decrease in the specific surface area from 988 m^2^/g to 471 m^2^/g occurs, while cumulative surface area reduces from 590 m^2^/g to 297 m^2^/g (Table 1). The observed reduction was about 50% after iron oxide impregnation. The cumulative pore volume showed a similar occupation (46%) compared to parent S-16. Monodispersed spherical silica HYPS show a type IV isotherm corresponding to the presence of mesopores. The silica hysteresis loop tends to be present at higher relative pressure of p/p_0_ >8. Among the three supports, spherical silica texture was lower with less surface area of 170 m^2^/g, pore volume of 0.35 cm^3^/g with intermediate average pore size distributions of 8.3 nm. After iron oxide loading over such lower texture HYPS, about 33% reduction in BET surface area was observed, while a significant increase in the pore size observed from 8.3 nm to 15.5 nm. MSU-F shows that iron oxide was inducted at the pores of foam leading to reduction in surface area (26% reduction) with an increase in open window type of pores (40 nm). The trend clearly shows the accumulation of iron oxide nanoparticles at the external pores of HYPS and MSU-F.

**Figure 2:**
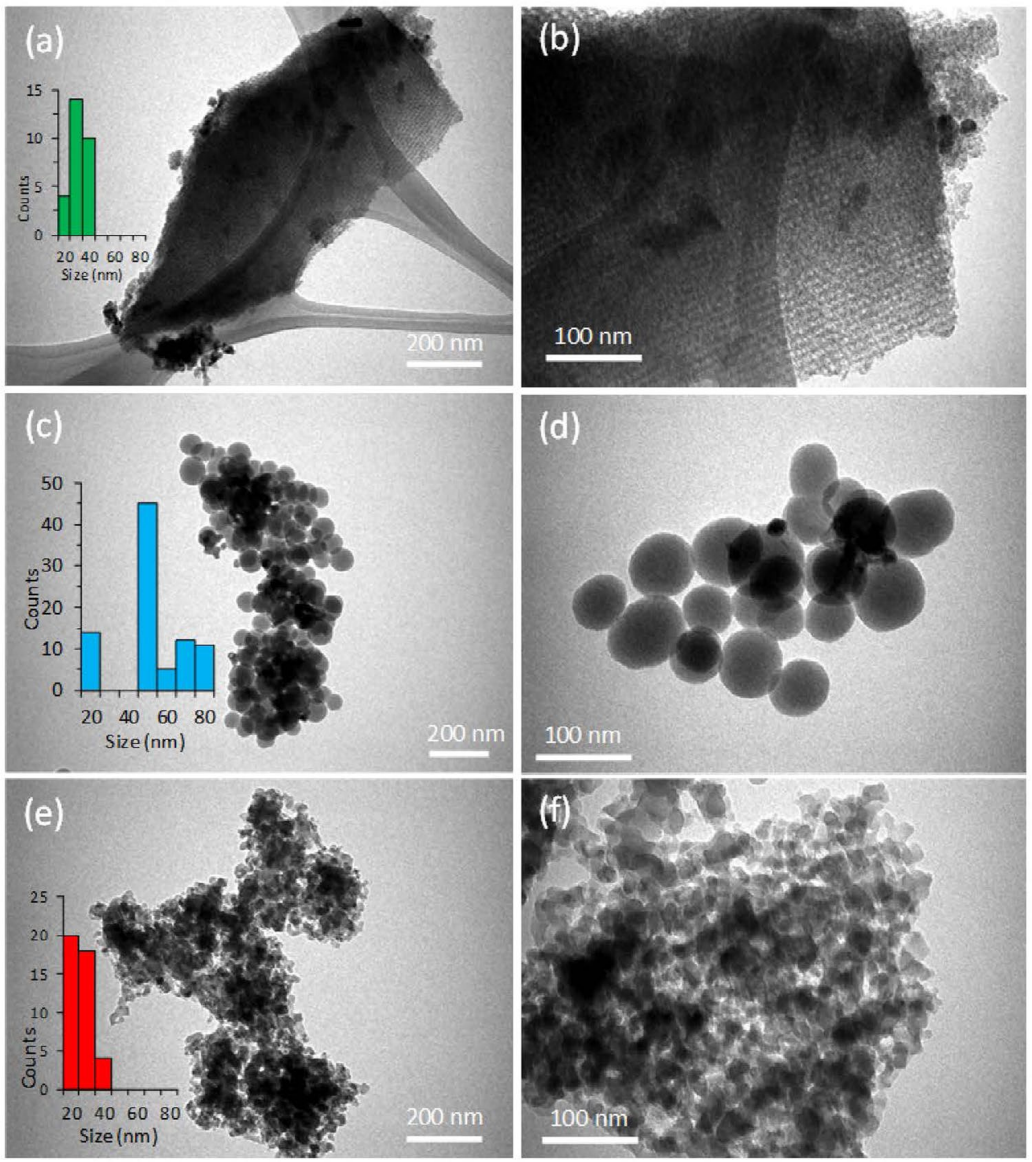
Surface morphology examined using TEM of as-prepared products; (**a**,**b**) 10 wt%SPIONs/S-16, (**c**,**d**) 10 wt%SPIONs/HYPS and (**e**,**f**) 10 wt%SPIONs/MSU-F.

The surface morphology of parent and 10wt.% SPIONs loaded silica S-16, HYPS, and MSU-F were analyzed through SEM (Fig. S3(a–f)). The parent S-16 was found to be composed of micron sized spherical spheres of about 4 µm. The iron oxide loading over S-16 showed no significant changes in the morphological characteristics compared to parent substance, which might be due to presence of high surface area (Fig. S3a,b). On the contrary, the 10 wt%SPIONs/HYPS showed the presence of monodispersed spherical silicas distributed uniformly in the range of about 80 nm. In case of 10 wt%SPIONs/HYPS, the presence of hetero nano-sized clusters were observed to be spread over spherical silica (Fig. S3c,d). In case of MSU-Foam, presence of agglomerated silica in irregular forms were observed. 10 wt%SPIONs/MSU-F showed some porous morphological characteristics changes with agglomerated nanospheres structures at lower scale bar of 3 μm (Fig. S3e,f). The TEM analysis at two different magnification scale bar 100 and 200 nm shows that SPIONs deposition are unique and depends on the nature of support, where the dispersion and agglomeration vary based on the nanocarriers pore architecture (Fig. 2(a–f). For instance, with three-dimensional cage type of SBA-16 pores, the presence of agglomerated forms of SPIONs as nanoclusters were observed along the pore channels (Fig. 2a,b). The presence of cage type of porous layer of SBA-16 appeared to be homogeneous with a constant thickness, and particles were found connected to the layers. In the case of 10 wt%SPIONs/HYPS, presence of uniform spherical silica was clearly observed, where a clear external agglomeration of SPIONs as nanosize clusters was observed to be distributed over spheres (Fig. 2c,d). In case of 10 wt%SPIONs/MSU-F, the presence of nanosized Fe_3_O_4_ was observed to be spread across the silica foam (Fig. 2e,f). The average particle size distribution of SPIONs over three supports were calculated (Fig. S4a–d). The particles were divided into different groups based on their size; 0–20, 21–30, 31–40, 41–50, 51–60, 61–70 and 71–80 nm. At constant loading (10 wt%), the nanoparticle size distribution of SPIONs was in the following order: HYPS > Si-SBA-16 > MSU-Foam. The average SPIONs particle size of the 10 wt%SPIONs/HYPS measured from TEM images was found to be high of about 51.0 ± 1.07 nm, followed by S-16 support in the range of 17 ± 1.83 nm. In support MSU-F, small sized agglomerated SPIONs in the range of 13 ± 0.92 nm was observed.

**Figure 3:**
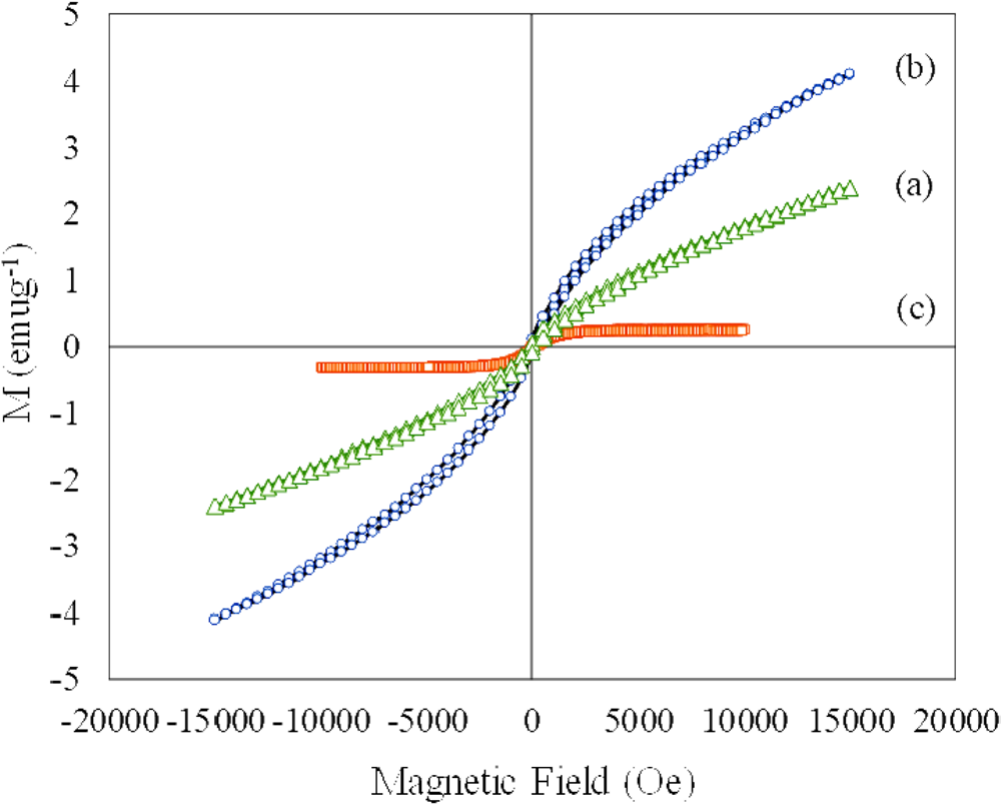
Vibrating sample magnetometer analysis of (**a**) 10 wt%SPIONs/S-16, (**b**) 10 wt%SPIONs/HYPS, and (**c**) 10 wt%SPIONs/MSU-F.

**Figure 4:**
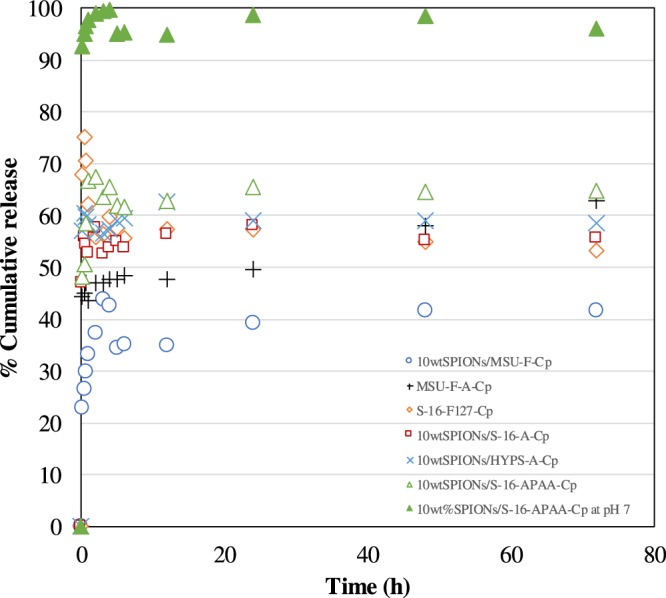
Drug release profile of different nanoformulations S-16, HYPS, MSU-F loaded with 10 wt% SPIONs, APTES, polyacrylic acid, copolymer F127, cisplatin in pH 5 and selected 10 wt%SPIONs/S-16-APAA-Cp in pH 7 at 37 °C for 72 h.

The magnetic properties of designed nanoparticle composites were measured using vibrating sample magnetometer (VSM). Figure 3(a–c) shows the VSM of (a) 10 wt%SPIONs/S-16 (b) 10 wt%SPIONs/HYPS and (c) 10 wt%SPIONs/MSU-F. 10 wt% SPIONs over three different structured silica showed different magnitude of superparamagnetization. The SPIONs magnetization generated over S-16, HYPS and MSU-F was in the following order: HYPS (4.08 emug^−1^) > S-16 (2.39 emug^−1^) > MSU-F (0.23 emug^−1^). It has been shown that presence of small sized nanoclusters at the walls of hexagonal shaped MCM-41 tends to form super paramagnetic interactions among Fe^3+^ species, while large nanoclusters contribute towards ferromagnetic property^18^. In the present study, an enforced impregnation of iron oxides into the mesocellular foam consisting large window pore sizes tends to generate such small nanosized Fe^3+^ clusters. As evidenced from TEM study (Fig. S4), the average nanoparticle of SPIONs over MSU-F was of 13 nm. In line with TEM analysis, the VSM spectra of MSU-F showed super paramagnetic behavior with narrow hysteresis. 10 wt% SPIONs impregnation over S-16 consisting ink bottle shaped pores generates medium nanoclusters with average particle size of 17 nm (Fig. 4a). SPIONs loading over monodisperse spherical silica HYPS generated the highest magnetization, while formation of large nanoclusters with average particle of 51 nm, tends to broaden the hysteresis loop indicating a shift towards ferromagnetic behaviour compared to MSU-F (Fig. 3b,c).

FT-IR spectroscopy was used to investigate the complexation nature of Cp over 10 wt%SPIONs/S-16 after functionalization with silane and polyacrylic acid (Fig. S5a–e). Spectra analysis of Cp showed an intense amine stretching bands at 3218 cm^−1^ and 3281 cm^−1^, while a broad peak at 1640 cm^−1^ was attributed to the characteristic peak of Cp (Fig. S5a). Figure S5(b,c) showed the FT-IR profile of nanocarrier S-16 and S-16 after iron oxide impregnation followed by silane functionalization (10 wt%SPIONs/S-16-A). In case of 10 wt%SPIONs/S-16-A-Cp (d) and 10 wt%SPIONs/S-16-APAA-Cp (e), the presence of weak amine band showed the conjugation between silane NH_2_ and Cl bond of Cp. Importantly, bending vibration at 1640 cm^−1^ indicates the functionalization of Cp and reaction between silica and Pt^II^ complex (Fig. S5d,e).

The Cp adsorption efficacy was studied for three nanoformulations 10 wt%SPIONs/S-16-A-Cp, 10 wt% SPIONs/HYPS-A-Cp and 10 wt%SPIONs/MSU-F-Cp. Among three functionalized nanocarriers, 10 wt%SPIONs/S-16-A-Cp showed the highest Cp adsorption of 91%. 10 wt% SPIONs/HYPS-A-Cp showed similar adsorption capacity to that of S-16 nanocarrier. The observed pattern indicated the increased solubility of drug at accessible functional groups located at cubic cage type pores of S-16 and spherical pores of HYPS. 10 wt%SPIONs/MSU-F-Cp showed slightly a lesser Cp adsorption of 86%.

The drug release of profile of various SPIONs/nanocarriers/Cp nanoformulations were studied using simulated tumor acid pH condition (pH 5) at 37 °C for 72 h (Fig. 4). Among the different nanoformulations, 10 wt%SPIONs/S-16-APAA-Cp showed highest Cp release of 65% for 72 h. In case of 10 wt% SPIONs/HYPS-A-Cp, a reasonable Cp release (58%) was observed followed by S-16 support. The result indicates that HYPS can be the other potential nanocarrier and can be exploited for drug delivery application. Mesocellular foam (MSU-F-A-Cp) consisting large widow size pores functionalized with APTES showed Cp release of about 63% at 72 h. However, 10 wt% SPIONs/MSU-F-Cp showed the lowest percentage cumulative release of 41% at 72 h. The trend signifies the importance of high textural properties of nanocarrier S-16, which helps to accommodate SPIONs, functionalization with APTES, polyacrylic acid and to release Cp (Table 1). The sustainable release of Cp shows the effectiveness of neutral type of silane APTES that will be charged positive at low pH (acidic condition) and proposed to coordinate with chloride ligand of Cp and help to attain plateau with time. The release trend clearly shows the immediate release formulation rather than burst release that can be aptly used for acute drug delivery targeting diseases. However, Cp over difunctional copolymer functionalized SBA-16 (S-16-F127-Cp) clearly showed an initial burst release of 75% at about 30 min, which then reduces to 60% at 72 h. Such pattern shows the inability of non-ionic polymers to coordinate with Cp at the surface of S-16. The order of Cp release among different nanoformulations are in the following order: 10 wt% SPIONs/S-16-APAA-Cp > 10 wt% SPIONs/HYPS-A-Cp > 10 wt%SPIONs/S-16-A-Cp > S-16-F127-Cp > MSU-Foam-A-Cp > 10 wt%SPIONs/MSU-F-Cp.

In order to study the oral route Cp delivery, pH variation study was performed simulating the strongly acidic pH of intestine and weak basic condition of colon. The capping property of 10wtFe/S-16-APAA-Cp was verified with the Cp release profile at pH 7 (Fig. 4). Our results indicate that 10wtFe/S-16-APAA-Cp showed an exemplary drug release capability (98%) at pH 7. The Cp release decreases from 55% at pH 5 to 30% at acidic pH of 2 over 72 h. The observed trend revealed that at low pH, the release ability of Cp is controlled by the electrostatic interactions between Cp/S-16 and acidic condition. Based on the earlier reported study at lower acidic pH = 5.6, the COOH group of polyacrylic acid is shown to collapses and forms intramolecular hydrogen bond in between COOH groups. Such behavior of polymer is reported to safeguard DOX by blocking inside the pore channels through encapsulation technique^19^. On the other hand, at pH 7.0, ionization of acid group of polyacrylic acid occurs leading to the formation of -COO- anions. Anion repulsion process was reported to swell the core of polymer, eventually leading to expansion of core and release of Cp. Polyacrylic acid complexation with Cp was reported to be critical for sustained release of Cp (80–90%) at two different pH conditions (7.4 and 5)^20^. In the present study, Cp release was significantly high of 98% at pH 7. The trend clearly indicates the presence of nano Cp at the cage type of pores of SBA-16, easily accessible to dissolved chloride solution. XRD analysis showed an increased Cp dissolution with S-16 support (Fig. S1). FT-IR confirms the complexation phenomenon of Cp with functionalized S-16 through ligand exchange process with available chloride ion (Fig. S5) thereby facilitating high Cp release from pH 2 to 7. Therefore, the stable release profile was attributed to the complexation phenomenon with copolymer through ligand exchange process with available chloride ion in physiological solution.

### *In vitro* anti-cancer study

**Figure 5:**
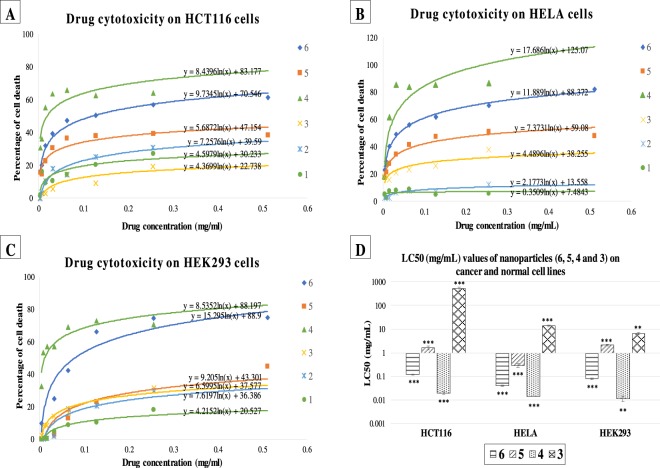
Cytotoxic effect of (1) S-16 (G-I), (2) SPIONs (G-II), (3) 10 wt%SPIONs/S-16 (G-III), (4) cisplatin (G-IV), (5) 10 wt%SPIONs/S-16-A-Cp (G-VI) and (6) 10 wt%SPIONs/S-16-APAA-Cp (G-V) on cancer and normal cell lines. (**A**) HCT116, (**B**) HeLa, (**C**) HEK293. These graphs were plotted to obtain LC_50_ values for each of the drugs tested on different cell lines (refer to Table 1). (**D**) Plotting the LC_50_ of effective drugs against the three cell lines (*p < 0.05, **p < 0.01 and ***p < 0.001, n = 3). Different concentrations of each nanoformulated drug and support were applied on each cell line then the cytotoxicity was measured by MTT assay, the resulted day was plotted in excel and calculated the LC_50_ using the equation of the logarithmic trendline. The p value and standard deviation were measured by one way ANOVA in GraphPad prism software.

The LC_50_ values varies between the drug Cp and nano formulated drugs (Group-IV, Group-V, Group-VI and their controls (Group-I, Group-II and Group-III) (Fig. 5). The LC_50_ of Group-I, Group-II and Group-III were very high (Table 2) indicating their zero effect in the cell killing when they included in the nanoparticles construction. HeLa cells were the most sensitive cell line towards Group-IV, Group-V, and Group-VI. The sensitivity of HeLa cells toward Group-VI is approximately 7.5 folds higher than its sensitivity toward Group-V, Whereas its more sensitive toward Cp by 3 folds than Group-VI. In HCT116, the sensitivity towards Group-VI was approximately 13.2 folds higher than its sensitivity toward Group-V, Whereas it was more sensitive toward Cp by 6.3 folds than Group-VI. In normal cell line HEK293 the cells were more resistant toward Group-V, the sensitivity to Group-VI was 69.5 folds higher than Group-V, but less by 9.5 folds than Cp. HEK293 showed a resistant toward Group-V compare to HCT116 and HeLa cancer lines. The effect of Group-VI on HCT116 and HEK293 were same but its more effective by 2.9 folds on HeLa cells.

**Table 2:** LC_50_ values for 10 wt%SPIONs/S-16, 10 wt%SPIONs/S-16-APAA-Cp, 10 wt%SPIONs/S-16-A-Cp, Cp, S-16 and SPIONs (Fe_3_O_4_) on cancer and normal cell lines.

Group	Nanoformulations	HCT116			HeLa			HEK293		
		LC_50_ (mg/ml)	p value	SD	LC_50_ (mg/ml)	p value	SD	LC_50_ (mg/ml)	p value	SD
G-1	S-16	75.461	<0.0001	0.9893	4.17E + 52	0.0397	1.313	1087.960	0.2903	2.572
G_II	SPIONs	4.179	<0.0001	4.287	18575529	<0.0001	0.3958	5.970	0.3007	5.162
G-III	10 wt% SPIONs/S-16	4.02E + 6	0.0009	1.751	1.31E + 17	0.0007	6.003	9.181	0.0247	1.704
G-IV	Cp	0.020	<0.0001	1.334	0.014	<0.0001	0.7946	0.011	0.0307	1.629
G-V	10 wt%SPIONs/S-16-APAA-Cp	1.649	<0.0001	0.5003	0.292	<0.0001	2.648	7.350	0.0415	2.088
G-VI	10 wt%SPIONs/S-16-A-Cp	0.121	<0.0001	0.8339	0.040	<0.0001	0.2648	0.105	0.0058	0.6983

**Figure 6:**
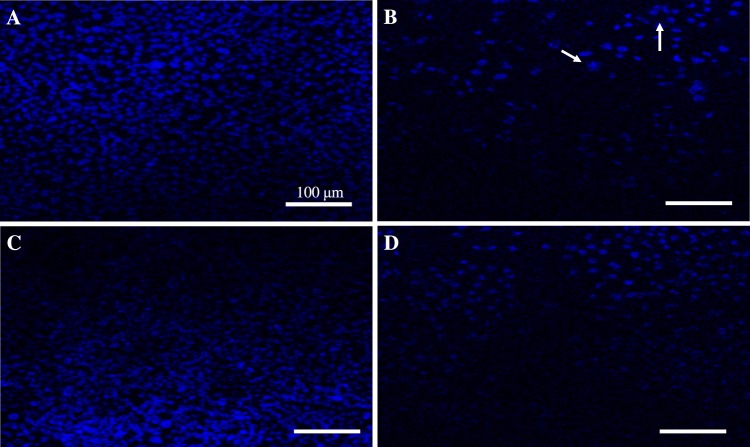
DAPI staining of treated HCT116 cells, the density stained areas are cell nuclei (blue color). (**A**) Control cancer cells. (**B**) Cisplatin treatment (G-IV), (**C**) 10 wt%SPIONs/S-16-APAA-Cp treatment (G-V)(0.188 mg/ml), (**D**) 10 wt%SPIONs/S-16-A-Cp treatment (G-VI) (0.188 mg/mL). All the scale bars are 100 µm. The arrow indicates the nucleus structural changes.

The apoptotic induction property of cisplatin (Cp), 10 wt%SPIONs/S-16-A-Cp, and 10 wt%SPIONs/S-16-APAA-Cp nanoformulations were studied on treating with HeLa and HCT116 cells using DAPI staining Fig. 6 shows the confocal microscopic images of treated HCT116. HCT116 cells were treated with Group-IV, Group-V and Group-VI for 24 h and stained with DAPI. This study elucidated the anti-cancer effect of nanoformulation based drug on nuclear condensation of cancer cells. Study showed reduction in cell number compare to untreated cancer cells. HCT 116 treated with Group-V, which consists SPIONs, APTES, polyacrylic acid showed no structural changes in the nucleus of HCT116 cells compared to the untreated cancer cells but showed significant reduction in cell number. Cp is a well-known chemotherapeutic drug used for treatment of many cancer types such as breast cancer and ovarian cancer showed DNA fragmentation and high reduction in cell number. However, Group-VI, which consists SPIONs, APTES and Cp interestingly decreased the quantitation of HCT116 cells and showed disintegrated nuclear morphology. These results indicate that Group-VI based nanoformulation is effective against colorectal cancer.

**Figure 7:**
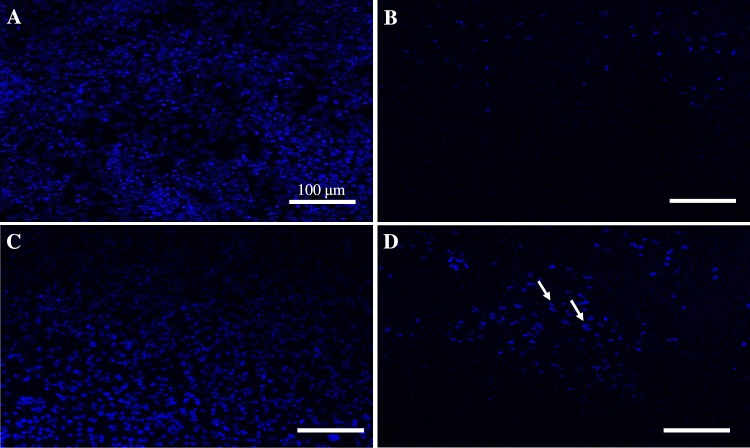
DAPI staining of treated HELA cells, the density stained areas are cell nuclei (blue color). (**A**) Control. (**B**) Cisplatin treatment (G-IV) (0.0342 mg/ml) (**C**) 10 wt%SPIONs/S-16-APAA-Cp treatment (G-V) (0.188 mg/ml), (**D**) 10 wt%SPIONs/S-16-A-Cp treatment (G-VI) (0.188 mg/ml). All the scale bars are 100 µm.

Figure 7 shows the confocal microscopic images of treated HeLa cells. The cells were treated with Group-IV, Group-V and Group-VI for 24 h and stained with DAPI to evaluate the effect of Group-VI on HeLa cells. Group-V showed a slight reduction in cell quantification compared to control cells. As expected Cp (Group-IV) killed 90% of HeLa cells since it used to treat cervical cancer by inhibiting the replication of DNA. However, HeLa cells proliferation was dramatically affected by Group-VI treatment with clear DNA fragmentation. This indicate that HeLa cells were more sensitive to Group-VI than HCT116 cells.

**Figure 8:**
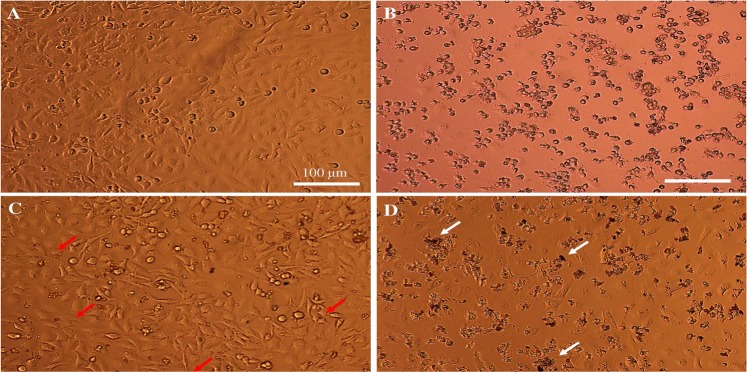
Cell morphology of treated Hela cells. (**A**) Control, (**B**) Cisplatin treatment (G-IV) (0.0342 mg/ml) (**C**) 10 wt%SPIONs/S-16-APAA-Cp treatment (G-V) (0.188 mg/ml), (**D**) 10 wt%SPIONs/S-16-A-Cp treatment (G-VI) (0.188 mg/ml). Whit arrow show cell debris and red arrow shows the SPIONs inside the cells. All the scale bars are 100 µm.

Figure 8 shows the cell morphology of treated HeLa cells using inverted microscope. The morphological pattern of treated HCT116 with Group-IV showed that almost all cells were completely dead after 24 h. Treatment of HCT 116 with Group-V showed the entry of the SPIONs particles inside the cells, which were indicated by the characteristics iron red to brown color. However, the presence of cells without kill indicates the poor release of Cp, which might be due to presence of pH sensitive polyacrylic acid. Whereas, in the Group-VI treatment, more than 80% of the cells are found to be dead. The presence of SPIONs were not easy to detect inside the cells indicating that the cells were taken in SPIONs, S-16, APTES, Cp nanoformulation (Group-VI) and were dead as the number of cells decreased dramatically. This indicate the effective release of Cp in Group-VI rather than in the presence of polyacrylic acid at pH 7.4.

**Figure 9:**
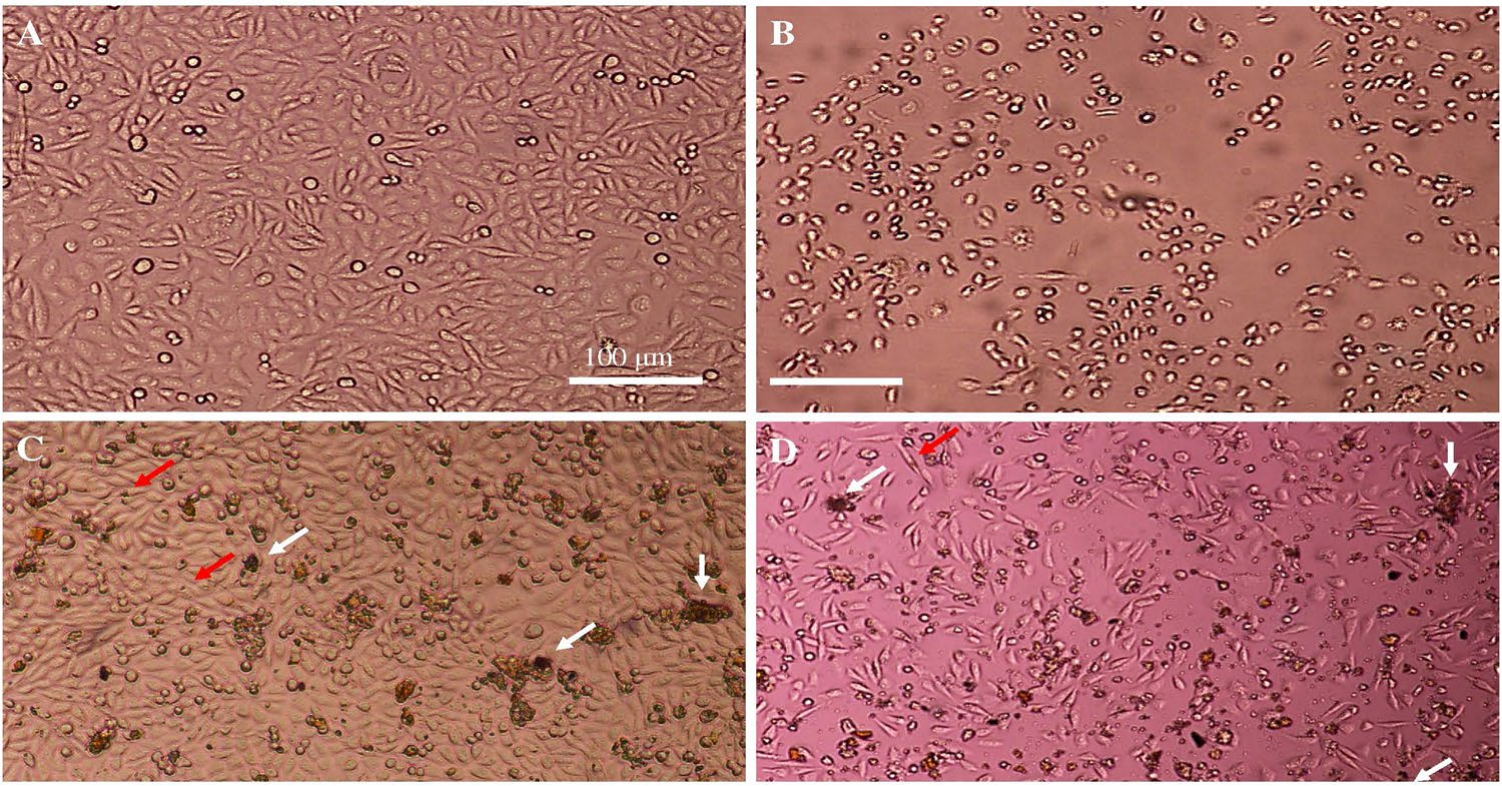
Cell morphology of treated HCT116 cells. (**A**) Control, (**B**) Cisplatin treatment (G-IV) (0.0342 mg/ml) (**C**) 10 wt%SPIONs/S-16-APAA-Cp treatment (G-V) (0.188 mg/ml), (**D**) 10 wt%SPIONs/S-16-A-Cp treatment (G-VI) (0.188 mg/ml). Whit arrow show cell debris and red arrow shows the SPIONs inside the cells. All the scale bars are 100 µm.

**Figure 10:**
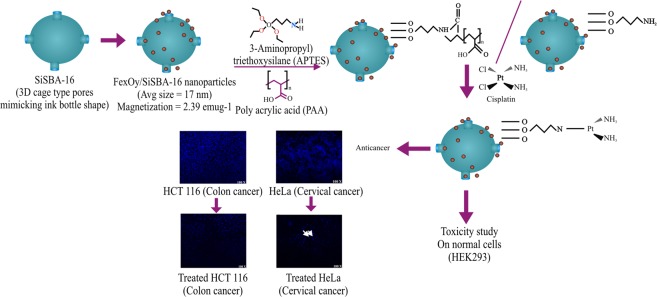
Drug delivery model for multifunctional 3D cage type S-16 loaded with 10 wt%SPIONs functionalized with APTES, polyacrylic acid and cisplatin.

To visualize the effect of different drug combination on cells morphology and number. cells were pictured before and after treatment using inverted microscope (Fig. 9). The control cells showed normal healthy cells, whereas, cells treated with Cp were almost completely dead after 24 h. In Group-V treatment, the cell debris was noticed as well as the entry of SPIONs into the cells (red arrow). But with very low release of Cp from SPIONs, S-16, APTES, polyacrylic acid nanoformulations, only 10% reduction of cell number occurs. Whereas, the Group-VI treatment was more effective and resulted in more than 60% of dead cells indicating effective release of Cp from SPIONs, S-16, APTES nanoparticle formulations. The images are visible evidence that clearly show the cell permeation by brown colored SPIONs impregnated S-16 nanocarrier and cell death shows the release of cisplatin. Overall, the high Cp adsorption efficiency, sustained pH stimuli drug release was advantage of 10 wt%SPIONs/S-16-Cp nanoformulation. The anticancer efficiency of 10 wt%SPIONs/S-16-Cp in cancerous cell lines (HeLa and HCT116) can be the most suitable for multifunctional stimuli responsive nanotherapeutics (Fig. 10).

## Conclusion

In this study, we optimized the multifunctional magnetic nanoformulation, SPIONs/S-16 to deliver Cp to cancerous cells in the biological media. 10wt.% SPIONs have been loaded over three silica based supports: S-16, HYPS and MSU-F. XRD analysis showed the presence of Fe_3_O_4_ in cubic structure of magnetite. By TEM analysis, average diameter of SPIONs was found about 51.0 ± 1.07 nm for SPIONs/HYPS, followed by S-16 in the range of 17 ± 1.83 nm. In case of MSU-F, a small sized agglomerated SPIONs was observed in the range of 13 ± 0.92 nm. VSM analysis exhibits the magnetization over S-16, HYPS and MSU-F in the following order: HYPS (4.08 emug^−1^) > S-16 (2.39 emug^−1^) > MSU-F (0.23 emug^−1^). The functionalization of nanoformulation was done with Cp. 10 wt%SPIONs/S-16-APAA showed the highest Cp adsorption to 91%. The order of Cp release studied using dialysis membrane in PBS solution. Nanosized Cp in functionalized cubic ink-bottle shaped pores of S-16 exhibited high release ability than spherical pores of HYPS and large window size pores of mesocellular foam. Based on drug release study, the best nanoformulation SPIONs/S-16 was selected for *in vitro* study on cancer cells. Treatment of HeLa and HCT116 cancerous cell line with 10 wt%SPIONs/S-16-A-Cp (Group-VI) showed high cell killing activity in compare to 10 wt%SPIONs/S-16-APAA-Cp (Group-V) treatment and other controls indicating the ability of 10 wt%SPIONs/S-16-A-Cp (Group-VI) formulation to increase Cp release in the cancerous cells. Therefore, the LC_50_ of 10 wt%SPIONs/S-16-A-Cp (Group-VI) was the lowest compare to the control in all cell lines, cancerous (HeLa and HCT116) and normal (HEK293). These results indicate that 10 wt%SPIONs/S-16-A-Cp (Group-VI) based nanoformulation is effective against colorectal and ovarian cancers, but the sensitivity of cervical cancer (HeLa cell) to 10 wt%SPIONs/S-16-A-Cp (Group-VI) was higher than colorectal cancer (HCT116). The controlled drug release and *in vitro* results suggest that this formulation could be suited for acute drug delivery for treating targeted diseases. Overall, magnetically active S-16 with magnetic resonance imaging capability in diagnosis can be further validated in *in vivo* experimental research to provide new insight about cancer nanotherapeutics.

## Supplementary information

Supplementary file
