# Supplementary material for: SPIONs/3D SiSBA-16 based Multifunctional Nanoformulation for target specific cisplatin release in colon and cervical cancer cell lines
Source: Sci Rep. 2019 Oct 10;9:14523. doi: 10.1038/s41598-019-51051-w (PMC6787005; doi:10.1038/s41598-019-51051-w)
Supplement: Supplementary file 1 — Supplementary file [file 41598_2019_51051_MOESM1_ESM.docx]

**SPIONs/3D SiSBA-16 based Multifunctional Nanoformulation for target specific cisplatin release in colon and cervical cancer cell lines**

**B. Rabindran Jermy^a^*, Munther Alomari^b^ Vijaya Ravinayagam**^c^***, Sarah Ameen Almofty^c^, S. Akhtar^d^**, **Jesu Francis Borgio***^e^***, Sayed Abdul Azeez***^e^*

^a^*Department of Nano-Medicine Research, Institute for Research and Medical Consultations, Imam Abdulrahman Bin Faisal University, Dammam, Saudi Arabia.*

*^b^Department of Nano-Medicine Research, Imam Abdulrahman Bin Faisal University, Dammam, Saudi Arabia.*

*^c^Department of Stem Cell Research, Institute for Research and Medical Consultations, Imam Abdulrahman Bin Faisal University, Dammam, Saudi Arabia.*

*^d^Department of Biophysics Research, Imam Abdulrahman Bin Faisal University, Dammam, Saudi Arabia.*

*^e^Department of Genetic Research, Institute for Research and Medical Consultations, Imam Abdulrahman Bin Faisal University, Dammam, Saudi Arabia.*

**Corresponding author*

B. Rabindran Jermy and Vijaya Ravinayagam

e-mail: rjermy@iau.edu.sa (B.R. Jermy); vrnayagam@iau.edu.sa (V. Ravinayagam); Tel: +966 3330881

**Fig. S1.** X-ray diffraction pattern of (a) cisplatin and (b) Fe/S-16-APAA-Cp.

**Fig. S2**. BET surface area and pore size distributions of (a) Si-16, (b) 10wt%SPIONs/S-16, (c) HYPS and (d) 10wt%SPIONs/HYPS, (e) MSU-F, and (f) 10wt%SPIONs/MSU-F.

**Fig. S3.** Surface morphology of as-prepared products; (a, b) 10wt%SPIONs/S-16, (c, d) 10wt%SPIONs/HYPS and (e, f) 10wt%SPIONs/MSU-F examined by SEM at low (a, c & e) and high magnifications (b, d & f).

**Fig. S4.** Size histograms and average size estimation of as-prepared products using TEM images. (a) 10wt%SPIONs/S-16, (b) 10wt%SPIONs/HYPS and (c) 10wt%SPIONs/MSU-F. (d) The average size of the particles for each sample. The particles were divided into different groups based on their size; 0-20, 21-30, 31-40, 41-50, 51-60, 61-70 and 71-80 nm.

**Fig. S5**. FT-IR spectra of (a) Cisplatin (b) S-16 (c) 10wt%SPIONs/S-16-APTES, (d) 10wt%SPIONs/S-16-A-Cp and (e) 10wt%SPIONs/S-16-APAA-Cp.
